# Genomic quantitative real-time PCR proves residual disease positivity in more than 30% samples with negative mRNA-based qRT-PCR in Chronic Myeloid Leukemia

**DOI:** 10.18632/oncoscience.65

**Published:** 2014-07-23

**Authors:** Ilaria S. Pagani, Orietta Spinelli, Elia Mattarucchi, Cristina Pirrone, Diana Pigni, Elisabetta Amelotti, Silvia Lilliu, Chiara Boroni, Tamara Intermesoli, Ursula Giussani, Luigi Caimi, Federica Bolda, Renata Baffelli, Eleonora Candi, Francesco Pasquali, Francesco Lo Curto, Arnalda Lanfranchi, Fulvio Porta, Alessandro Rambaldi, Giovanni Porta

**Affiliations:** ^1^ Department of Experimental and Clinical Medicine, Insubria University, Varese, Italy; ^2^ Department of Experimental Medicine and Surgery, Tor Vergata University, Rome, Italy; ^3^ Hematology laboratory, USC Hematology, Papa Giovanni XXIII Hospital, Bergamo, Italy; ^4^ Laboratory of Medical Genetics, Papa Giovanni XXIII Hospital, Bergamo, Italy; ^5^ Department of Molecular and Translational Medicine, University of Brescia, Brescia, Italy; ^6^ Laboratory of chemical-clinical analysis, Section of Hematology and blood coagulation, Stem Cells laboratory, Spedali Civili of Brescia, Brescia, Italy

**Keywords:** chronic myeloid leukemia, minimal residual disease, stop imatinib, leukemic stem cells, DNA Q-PCR

## Abstract

Imatinib mesylate (IM) is the first line therapy against Chronic Myeloid Leukemia, effectively prolonging overall survival. Because discontinuation of treatment is associated with relapse, IM is required indefinitely to maintain operational cure. To assess minimal residual disease, cytogenetic analysis is insensitive in a high background of normal lymphocytes. The qRT-PCR provides highly sensitive detection of BCR-ABL1 transcripts, but mRNA levels are not directly related to the number of leukemic cells, and undetectable results are difficult to interpret. We developed a sensitive approach to detect the number of leukemic cells by a genomic DNA (gDNA) Q-PCR assay based on the break-point sequence, with a formula to calculate the number of Ph-positive cells. We monitored 8 CML patients treated with IM for more than 8 years. We tested each samples by patient specific gDNA Q-PCR in parallel by the conventional techniques. In all samples positive for chimeric transcripts we showed corresponding chimeric gDNA by Q-PCR, and in 32.8% (42/128) of samples with undetectable levels of mRNA we detected the persistence of leukemic cells.

The gDNA Q-PCR assay could be a new diagnostic tool used in parallel to conventional techniques to support the clinician's decision to vary or to STOP IM therapy.

## INTRODUCTION

A decade ago the tyrosine kinase inhibitor (TKI) Imatinib mesylate (IM) provided a targeted therapy for patients with advanced CP, revolutionizing the management of CML; it now represents the first line therapy [1-7]. CML patients must, however, be monitored continuously to follow their response to IM and to verify that disease does not recur [8]. Monitoring relies mainly on cytogenetic techniques and quantitative real-time reverse transcriptase PCR (qRT-PCR) [1, 9-18].

Cytogenetic techniques are still standard to diagnose CML as they are widely available and reliable and can detect other chromosomal changes, but they are not very sensitive [19].

qRT-PCR is the most sensitive technique now available to monitor BCR-ABL1 chimeric mRNA levels after initial diagnosis and treatment. Results are expressed as the ratio of BCR-ABL1 transcript numbers to the number of control gene transcripts [20]. In 2006, the National Institutes of Health Consensus group proposed an international scale (IS) to standardize the results [21].

Despite the high sensitivity of the qRT-PCR, the technique has some limits related to the interpretation of undetectable results. The mRNA is susceptible to degradation and the efficiency of cDNA synthesis can vary [22], indeed the accuracy of the method depends critically on the ability of testing laboratories to measure absolute numbers of control gene transcripts in a comparable manner and to achieve the sensitivity required for the BCR-ABL1 detection [23,24]. Finally this technique detects only leukemic transcripts, which may not be necessarily proportional to the number of Ph-positive cells and completely misses transcriptionally silent cells. Thus, it may not be clear whether patients have achieved a truly “safe haven”, so that they can be taken off therapy [25].

This can be very important in treatment discontinuation trials. Indeed the IM discontinuation in patients achieving a complete molecular response is associated with molecular relapse in about 60% of patients [26,27]. Thus the current recommendation is lifelong treatment to maintain remission at considerable costs and with risk of long-term complications, reduced compliance and drug resistance [28].

We are proposing a sensitive approach to detect the number of leukemic cells directly, using a DNA-based biomarker specific for each patient. We developed a patient specific genomic DNA Q-PCR (g-DNA Q-PCR) assay based on the BCR-ABL1 genomic break-point and a formula to calculate the number of Ph-positive cells [29]. Here we expanded findings by monitoring CML patients from an early chronic phase up to 8 years of IM treatment, and we compared results with cytogenetic and mRNA analysis. Our study showed the presence of Ph-positive cells in 32.8% (42/128) of samples with undetectable levels of mRNA. Finally we applied our accurate alternative approach in the evaluation of BCR-ABL1 in CD34+ sorted cells, suggesting the persistence of leukemic stem cells.

## RESULTS

### Cytogenetic analysis: CBA and FISH

Eight CML patients in early CP were monitored for residual disease. The presence of the t(9;22) (q34;q11) translocation was evident in patients 1, 2, 4, 5, 6, 7, 8 at diagnosis. Karyotype analysis on patient 3 showed a rare t(9;22;16)(q34;q11;q24) translocation, and FISH confirmed the BCR/ABL1 signal at 22q11.2 as a result of a cryptic three-way rearrangement between chromosomes 9, 22 and 16. The FISH analysis in addition highlighted the ABL1 deletion in the derivative of chromosome 9 in patient 4.

A complete cytogenetic response (CCyR: No Ph-positive metaphases)[10,30] was achieved by 75% of patients (Pts.1, 2, 3, 4, 5, 6) within 6 months of treatment with Imatinib mesylate. Patient 7 was in partial cytogenetic response (PCyR: 1%-35% Ph-positive metaphases) [10,30] until sixth month of therapy, and then achieve CCyR. By contrast, CBA and FISH were normal at six months in patient 8, but leukemic cells were detected by CBA (1/22 metaphases) and confirmed by I-FISH (3/500 nuclei) at twelve months. Stable CCyR was achieved at the eighteenth month of therapy after an increase of IM dose to 600 mg/day (Supplemental Table S1).

### Molecular monitoring assessed by qRT-PCR based on mRNA

The mRNA detection was performed by using a commercial kit approved for the clinical diagnosis (M-Bcr FusionQuant Standard Kit-Ipsogen, Stamford, USA). We analyzed by qRT-PCR 8 patients under IM therapy for an average period of 90 months with a total of 128 samples.

**Figure 1:**
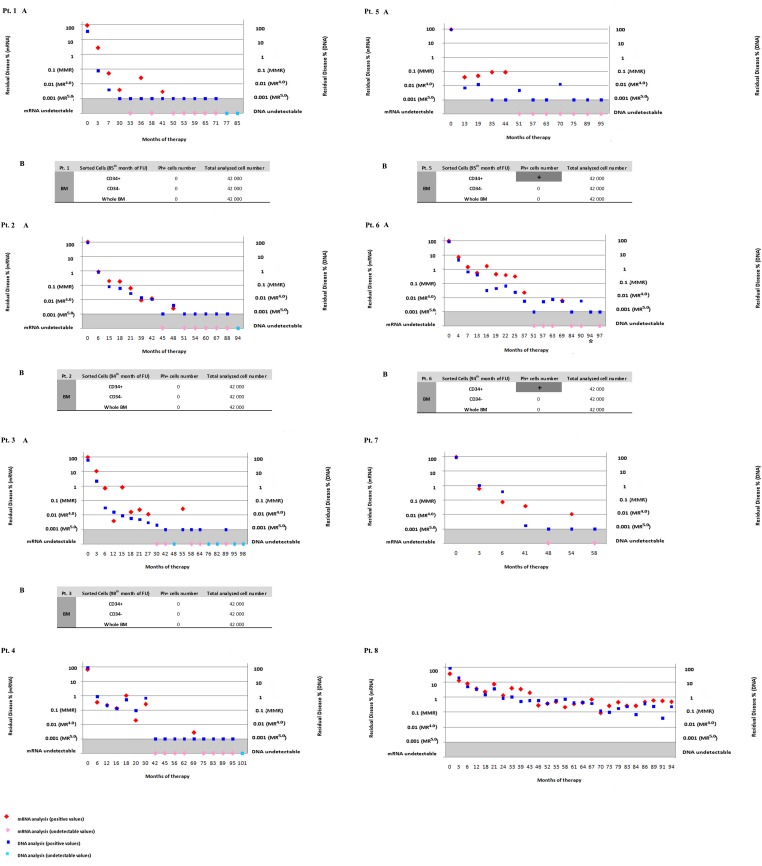
Residual disease assessed by qRT-PCR based on mRNA and Q-PCR based on gDNA A-The 8 panels show individual patients studied from diagnosis through 8 years of follow-up under IM therapy. On the x-axis are indicated the months of IM therapy. On the y-axis to the left we represented the percentage of MRD assessed by mRNA based qRT-PCR, and to the right the percentage of MRD assessed by gDNA based Q-PCR. Results of quantifying BCR-ABL1 transcripts are expressed as the ratio of BCR-ABL1 to ABL1 mRNA. The BCR-ABL1 expression of 0.1% corresponds to the standard baseline of the Major Molecular Response (MMR). MR4.0 is related to either detectable disease with ≤0.01% BCR-ABL1 IS, or undetectable disease in cDNA with ≥10 000 ABL1 transcripts. MR4.5 is related to either detectable disease with ≤0.0032% BCR-ABL1 IS, or undetectable disease in cDNA with ≥32 000 ABL1 transcripts. The same samples were tested by Q-PCR based on genomic DNA. We defined the “quantitative range” of detection as the part of the standard curve over which MRD levels can be quantified reproducibly and accurately, and we defined the “limit of sensitivity” as the lowest MRD level that could still be detected (although not reached in all replicates). The detection of MRD at the limit of sensitivity (0.001%) was indicated as positive but not quantified. In Pt. 6 at 94 months mRNA was not evaluable (*). B-The MRD was evaluated in CD34+ and CD34-sorted cells in patients 1, 2, 3, 5, 6. We analyzed the % of Ph+ cells by gDNA Q-PCR, and we then calculated the number of leukemic cells. The results were indicated as positive (+) but not quantified at the limit of sensitivity.

Patient 1, 2, 3, 4, 5 achieved mRNA undetectable levels for at least three years (range, 26-44 moths), while patient 6 and 7 did not show consistent undetectable values over the years, and patient 8 never even achieved major molecular response (MMR) demonstrating sub-optimal response to the therapy (Figure 1 and Supplemental Table S1).

### Sensitivity of gDNA Q-PCR

**Figure 2:**
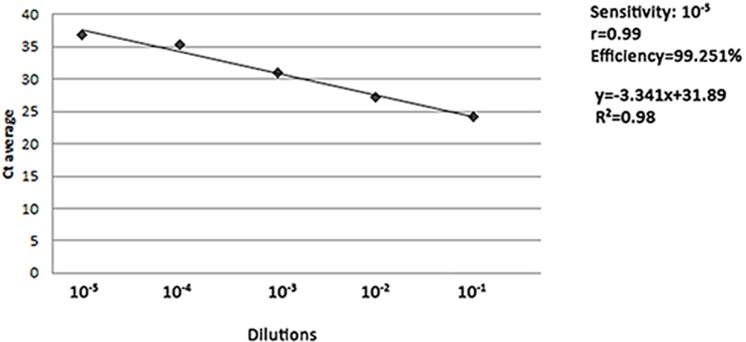
Sensitivity of Q-PCR assay Genomic DNA from K562 cell line was diluted with G147A commercial human male genomic DNA (BCR-ABL1 negative) to simulate different concentrations of leukemic DNA. The series covered a range of 5 logs of dilution of K562 gDNA from 10-1 to 10-5, according to accepted criteria of sensitivity. The dilutions were tested in 6 replicate reactions and the standard curve obtained by plotting the logarithmic value of the dilution (x-axis) against the average cycle threshold (Ct) of the reactions at each dilutions (y-axis). The efficiency of the reaction was calculated by the following formula: η=(10^−1/slope^−1)*100.

To test the sensitivity and the efficiency of the gDNA Q-PCR method we assessed 10-fold dilutions of K562 leukemic cells in normal genomic DNA, as described in Materials and Methods. The threshold of sensitivity was 10-5. We generated a standard curve over 5 logs of dilution, with a slope of −3.341 and a correlation coefficient (r) of 0.99, very close to the optimal theoretical slope of −3.32 and correlation coefficient of 1. The efficiency of the assay was of 99.251% (Figure 2).

### Comparison of residual disease assessment by qRT-PCR based on mRNA and gDNA Q-PCR

Minimal residual disease was monitored comparing the gDNA Q-PCR with the mRNA analysis. For each patient, a genomic assay was developed following the requirements for efficiency, sensitivity and absence of spurious amplifications as described in material and methods.

During the first years of therapy (average of 40 months) the percentage of residual disease assessed by gDNA Q-PCR and mRNA qRT-PCR showed similar trend, with values above the MMR; later, when leukemic cells decrease, the two assays dramatically diverged (Figure 1).

Positive levels of mRNA were found in 60.2% (77/128) of samples analyzed by qRT-PCR, whereas Ph-positive cells were detected by gDNA Q-PCR in 93% (119/128) of samples. These differences are statistically significant at p=0.0004 and t=4.627 (Figure 1 and Table 1).

**Table 1:** Comparison between positive values obtained by qRT-PCR based on mRNA and Q-PCR based on gDNA

	Patients	Number of Samples (N)	mRNA + (%, n/N)	mRNA − (%, n/N)	DNA + (%, n/N)	DNA − (%,n/N)	DNA+/mRNA − (%,n/N)
	1	15	40 (6/15)	60 (9/15)	86.7 (13/15)	13.3 (2/15)	46.7 (7/15)
	2	15	53.3 (8/15)	46.7 (7/15)	93.3 (14/15)	6.7 (1/15)	40 (6/15)
	3	19	47.4 (9/19)	52.6 (10/19)	73.7 (14/19)	26.3 (5/19)	26.3 (5/19)
	4	17	47.1 (8/17)	52.9 (9/17)	94.1 (16/17)	5.9 (1/17)	47.1 (8/17)
	5	12	41.7 (5/12)	58.3 (7/12)	100 (12/12)	0 (0/12)	58.3 (7/12)
	6	17	58.8 (10/17)	41.2 (7/17)	100 (17/17)	0 (0/17)	41.2 (7/17)
	7	7	71.4 (5/7)	28.6 (2/7)	100 (7/7)	0 (0/7)	28.6 (2/7)
	8	26	100 (26/26)	0 (0/26)	100 (26/26)	0 (0/26)	0 (0/26)
Tot	8	128	60.2 (77/128)	39.8 (51/128)	93 (119/128)	7 (9/128)	32.8 (42/128)

In all samples positive for chimeric transcripts we measured positive levels of corresponding genomic DNA, confirming the sensitivity of the gDNA Q-PCR method.

Undetectable levels of mRNA were found in 39.8% (51/128) of samples (Figure 1 and Table 1). It is interesting to note that among negative samples detected by mRNA analysis, we found DNA positivity by gDNA Q-PCR in 32.8% (42/128) of samples. These differences were statistically significant at p<0.0001 and t=6.544.

Finally, 7% (9/128) of samples were undetectable for both the techniques (Figure 1 and Table 1).

### Cell sorting and leukemic stem cell evaluation

We collected bone marrow from patients 1, 2, 3, 5 and 6 at the 85^th^, 94^th^, 98^th^, 95^th^ and 94^th^ month of follow-up respectively. We sorted CD34+ from bone marrow (BM), and negative fractions were also collected after population selection. We performed gDNA Q-PCR in order to detect BCR-ABL1 positive cells in all fractions.

Sorted cells from patients 1, 2 and 3 showed negativity for leukemic cells in all fractions, confirming the negativity of the PB, while the CD34+ fraction from patients 5 and 6 selected from negative BM showed gDNA positivity (Figure 1).

The mRNA analysis on sorted cell showed low levels of ABL1 control gene and the data were rejected because the gene expression was out of range.

## DISCUSSION

Therapy with TKIs is sufficient to prevent the progression to advanced CML and inhibit recurrence [31]. To maintain such “operational cure” IM is required indefinitely, despite financial cost, and considerable side effects [32]. The European Organization for Research and Treatment of Cancer (EORTC) developed questionnaires to assess the quality of life (QOL) in CML patients [33]. Phillips and colleagues analyzed QOL in patients taking IM compared to age-and gender-matched controls with no history of cancer. They concluded that TKIs have side effects and intolerance for many patients. Chronic grade I toxicities such as diarrhea may significantly impair QOL, and some patients choose not to take IM to avoid toxicity [34]. In fact, IRIS study reported that <10% of patients with 5 years of follow-ups stopped IM because of intolerable toxicity [32].

Recent studies therefore suggest the possibility to discontinue TKIs therapy for patients in complete molecular response (CMR) for at least 2 consecutive years [35]. The National Comprehensive Cancer Network (NCCN) guidelines and the European LeukemiaNet (ELN) recommendations define CMR as undetectable levels of BCR-ABL1 fusion transcripts detected by qRT-PCR [23]. The French multicenter non-randomized Stop IM (STIM) trial identified a sub-set of CP-CML patients with durable CMR for 2 years who could discontinue the therapy [26]. 40% of patients who achieved CMR remained disease-free after drug discontinuation, suggesting this subgroup of patients have reached a real cure [26].

However, 60% of patients in the STIM study relapsed [26], so the safe introduction of a withdrawal policy require a reliable method to identify those patients with the highest probability of relapse. The likelihood of relapse post-withdrawal is related to the persistence of residual disease, which may include the presence of transcriptionally silent leukemic stem cells [36,37], at a level that is below the threshold of sensitivity by the standard qRT-PCR [38]. The sensitivity of the qRT-PCR depends on the ability of the laboratories both to measure absolute numbers of ABL1 control gene transcripts in a comparable manner, and to achieve the sensitivity required for the BCR-ABL1 detection [23,24, 39-41].

Thus the accurate determination of residual disease is important for the identification of patients that could stop the therapy.

We propose the gDNA based Q-PCR as new sensitive diagnostic tool to directly detect the number of leukemic cells independently from their transcriptional status. This assay based on the sequencing of the genomic breakpoint [29,42] permits a pragmatic definition of the limit of quantization and sensitivity to evaluate minimal residual disease (MRD), as described by guidelines for the detection of MRD by genomic Q-PCR in acute lymphoblastic leukemia (ALL) [43]. We are able to calculate the exact number of leukemic cells when the MRD fell within the range of quantization, and to detect MRD as positive but not quantifiable at the limit of sensitivity. Finally in order to better understand the pathogenesis of CML we separated different cell populations from bone marrow. The analysis of cells sub-fractions could also be very useful when we are at the limit of the sensitivity of the method, in order to enrich Ph-positive cells.

We confirmed the sensitivity of the Q-PCR technique, measuring positive levels of gDNA in all BCR-ABL1 mRNA positive samples.

Of note, we detected Ph-positive cells by gDNA Q-PCR in 32.8% (42/128) of the samples with mRNA negativity, and we never found samples negative by gDNA Q-PCR in the presence of mRNA positivity. All mRNA analysis were performed following the standard operating procedures of the Lab Net and GIMEMA CML Working Party. The mRNA negativity not due to degradation could probably reflect either a limit of detection of the method, or the presence of transcriptionally silent leukemic cells.

According to the current criteria of STOP IM protocol, patients 1, 2, 3, 4 and 5, with undetectable levels of mRNA for more than 2 years, could be possible candidates to discontinue the therapy. However, patients 1, 2, 3, 4 become negative for gDNA Q-PCR only at the last FUs. This result was confirmed by the negativity of the CD34+ sorted cells. On the contrary, we demonstrated that patient 5, negative for mRNA, showed gDNA positivity in all FUs. The additional presence of Ph-positive leukemic cells in the CD34+ sub fraction at 95 months of therapy could indicate a prediction of the relapse.

In 2005 Michor and colleagues asked whether IM could eradicate leukemia stem cells. They analyzed the dynamics of response of leukemic cells to IM, and after it was discontinued [44]. They observed a biphasic decline of leukemic cells and a relapse after drug discontinuation. Hence they concluded that long-term IM treatment does not completely deplete the cell population that drives the disease [25, 44-47]. It has been proposed that CML stem cells use survival signals other than BCR-ABL1 kinase to maintain their viability in the presence of tyrosine kinase inhibitors. This suggests that CML stem cell elimination may require completely different strategies such as targeting stem cell self-renewal or disrupting interactions with the microenvironment, and the gDNA technique could demonstrate its utility in monitoring Ph positive cells at every stage of differentiation.

Most likely, growth arrest and apoptosis signals modulate the biological outcomes of BCR-ABL inhibition [45]. Different strategies are proposed for overcoming IM resistance, such as blocking cytokine signaling or using Rapamycin or other mTOR inhibitors [48-50].

New therapeutic approaches are based on the use of demethylating agents, such as 5′-azacytidineand 4-phenilbutyrate, that leads to a decrease of BCR-ABL1 expression and to a decrease in the proliferation rate of Ph+ human CML cell lines [51,52]. Several reports correlate CML with the DNA methylation and new insights suggest the involvement of epigenetic dysregulation of miRNAs in leukemogenesis [53-55]. The silencing and the down-regulation of miR-15/16 and miR-31/155/564 are involved in the pathogenesis of CML [53]. Of significant interest, the epigenetic silencing of the tumor suppressor miR-203 enhances the expression of the direct targets ABL1 and BCR-ABL1 [56]. Restoration of the silenced miR-203 expression, either directly or through the use of demethylating agents might represents a new therapeutic approach in CML [52]. A similar effect was interestingly observed after treatment with IM. The IM-induced demethylation of the promoter region of the miR-203, decreases the levels of BCR-ABL1 mRNA, and suppresses the growth of the BCR-ABL1 positive leukemic cells [53,57-68].

The down-regulation of the BCR-ABL1 mRNA induced by IM supports the importance of a new diagnostic tool independent from the transcriptional status of the cells under study. The gDNA based method is technically arduous since it requires the identification of the breakpoint and a customized assay that is patient specific. However, the gDNA is not susceptible to degradation as the mRNA, the patient-specific assay reduces the possible cross contaminations due to the presence of cDNA from another RNA, and the gDNA Q-PCR assay permits the identification of the transcriptionally silent leukemic cells. In addition because the primer set designed by the EAC to amplify ABL1 as control gene is located on exon a2, it may also amplify the BCR-ABL1 fusion gene transcript, underestimating the tumor load [20].

In conclusion in our 8 years trial we demonstrated the positivity of the gDNA Q-PCR in 32.8% of samples negative for the mRNA qRT-PCR. This technique in parallel with mRNA could be used to explain why some patients relapse and others do not, when IM is discontinued for brief periods [69,46,48,58]. The gDNA based Q-PCR coupled to the sorter of CD34+ cells could be used as a new diagnostic tool, by providing to clinicians additional informations about the pathogenesis of CML, the disease status and the response to therapy.

## MATERIALS AND METHODS

### Cell lines

K562 cells (CCL-243) were purchased from ATCC and cultured according to ATCC instruction.

### Patient samples and treatment regimens

We monitored 8 patients with CML diagnosed in the early CP for an average period of 90 months (range, 58-101 months), with a total of 128 samples. All patients showed BCR-ABL1 b2a2 fusion transcript. The patients included 6 men and 2 women, with a median age at diagnosis of 58 years (range, 47-70). The majority of patients were treated with IM monotherapy at a starting dose of 400 mg/day, except for patient 4, who was participating in an 800 mg/day trial (Table 2). The dose for patients 5 and 8 was increased to 600 mg/ day as a consequence of suboptimal cytogenetic findings observed at 12 and 6 months, respectively. Patients 5 and 8 continued therapy with 600 mg/day of IM until 73 and 44 months respectively, after which dosage was reduced to 400 mg/day; and patient 4 continued therapy with 800 mg/day until 64 months, when the dosage was reduced to 400 mg/day (Supplemental Table S1).

**Table 2:** Table of patients at study entry

Patient ID	Sex	Age (years)	Date of Diagnosis	Diagnosis	Translocation	Sokal Risk	Start of theraphy	Therapy (IM)
1	M	55	21/02/2006	CML	t(9;22) (q34;q11) p210 b2a2	Low	07/03/2006	400mg/die
2	M	57	20/05/2005	CML	t(9;22) (q34;q11) p210 b2a2	0,47	25/05/2005	400mg/die
3	F	60	30/05/2005	CML	t(9;22;16) (q34;q11;q24) p210 b2a2	0,37	08/06/2005	400mg/die
4	M	49	03/02/2005	CML	t(9;22) (q34;q11) p210 b2a2	0,82	14/02/2005	800mg/die
5	M	56	08/10/2004	CML	t(9;22) (q34;q11) p210 b2a2	0,86	12/10/2004	400mg/die
6	M	47	30/03/2005	CML	t(9;22) (q34;q11) p210 b2a2	0,55	12/04/2005	400mg/die
7	M	70	06/06/2005	CML	t(9;22) (q34;q11) p210 b2a2	0,73	17/06/2005	400mg/die
8	F	68	02/12/2004	CML	t(9;22) (q34;q11) p210 b2a2	0,64	21/12/2004	400mg/die

Patient 7 died after heart transplantation in 2010.

Informed consent for the use of cells for research was obtained in accordance with the Declaration of Helsinki and with approval of the Ethics Committee of the Insubria University and the Hospital of Bergamo, Italy.

### Cytogenetic analysis

Conventional cytogenetic analyses (chromosome banding analysis, CBA, and fluorescence in situ hybridization, FISH) were carried out routinely. All analytical procedures were subjected to quality control according to ISO 9001:2000 accreditation of the laboratory, and samples that did not conform to standards were rejected [29].

### qRT-PCR

All mRNA analysis were performed following the standard operating procedures of the Lab Net and GIMEMA CML Working Party.

Bone marrow and/or peripheral blood samples for qRT-PCR were obtained before therapy, then they were collected at 3, 6, 12 months after therapy, and thereafter every 6 months.

Total RNA was extracted with the RNeasy Mini Kit (Qiagen, Milano, Italy) from blood and bone marrow samples previously treated with the HetaSept gradient (Stem Cell Technologies, Vancouver, Canada) to eliminate red cells and erythroid precursors. The RNA integrity was tested by 2100 Bioanalyzer Instrument (Agilent Technologies, Santa Clara, CA) after each extraction, and quantified by nanodrop spectrophotometer (Thermo Scientific, Wilmington, USA). RNA (1 μg) was reverse transcribed using high efficiency Superscript III reverse-transcriptase (Invitrogen by Life Technologies, Monza, Italy) following the EAC procedure [70,71].

Transcripts were characterized following the protocol proposed by van Dongen and colleagues [72] b2a2 junctions were identified in patients, and b3a2 in K562 cell line.

qRT-PCR was performed using 200 ng of RNA and the M-Bcr FusionQuant Standard Kit (Ipsogen, Stamford, USA) according to the manufacturer's protocol, on the ABI PRISM 7700 Sequence Detector System (Perkin Elmer, Massachusetts, USA). The kit is approved by the European Against Cancer (EAC) group for in vitro diagnostic and it contains the same primers and probe used by the EAC to quantify BCR-ABL1 and ABL1 transcripts, plus a series of control and calibrators. ABL1 was used as a housekeeping gene to correct differences in RNA quality and/or reverse transcription efficacy. BCR-ABL1 and ABL1 plasmid dilutions were used as standards, and the final results were calculated as the ratios of BCR-ABL1 to ABL1 and expressed in percentages. All experiments were performed in triplicate for BCR-ABL1 and in duplicate for ABL1, and results were expressed as percent ratio to ABL1. The BCR-ABL1/ABL1 ratios were further multiplied by the conversion factor of the Bergamo laboratory to set the results on an international scale (IS). Samples yielding an ABL threshold cycle greater than 29, corresponding to less than 1000 ABL1 transcript copies, were considered as having degraded RNA and discarded. To test the robustness of the quantitative method efficiency, sensitivity and reproducibility were tested. The regression curve need an average slope of −3,32 (range −3,20 and −3,60) with R2=1.

The molecular response is assessed according to the IS as the ratio of the BCR-ABL1 transcripts to the ABL1 transcripts. Results are expressed as BCR-ABL1% on a log scale, where 10%, 1%, 0,1%, 0,01%, 0,0032% and 0,001% correspond to a decrease of 1, 2, 3, 4, 4.5 and 5 logs respectively. The BCR-ABL1 expression of ≤0.1% corresponds to the standard baseline of the Major Molecular Response (MMR). The deep molecular response MR4.0 is related to either detectable disease with ≤0.01% BCR-ABL1 IS, or undetectable disease in cDNA with ≥10 000 ABL1 transcripts. The MR4.5 is related to either detectable disease with ≤0.0032% BCR-ABL1 IS, or undetectable disease in cDNA with ≥32 000 ABL1 transcripts. The term complete molecular response (CMR) is substituted with the term molecularly undetectable leukemia, with specification of the number of the control gene transcripts copies [23,24].

The sensitivity achieved in all sample was of MR4.5.

### BCR-ABL1 DNA breakpoint detection

Genomic BCR-ABL1 fusion sequences (Supplemental Table S2) were previously characterized in our laboratory through a system originally developed for genome walking [42]. In the same paper, a detailed explanation of the sequencing protocol was reported. Briefly, the DNA from patients was extracted from bone marrow, fragmented, ligated to adaptors, and amplified by nested PCR using a BCR specific forward primer. Thus, the sequence of genomic breakpoints was assessed.

### Patient-specific gDNA Q-PCR for genomic breakpoint

Genomic DNA (gDNA) was extracted from the same samples used for molecular monitoring of mRNA. Peripheral blood or bone marrow cells were collected by centrifugation on a Hetasept gradient (Stemcell technologies). Mononuclear cells were resuspended in guanidine isothiocyanate solution and stored at −80°C. DNA was then extracted and purified with All-Prep DNA/ RNA kit (Qiagen, Milano, Italy). The concentration of gDNA was determined by Qubit fluorometer (Invitrogen by Life Technologies, Monza, Italy) and its integrity checked by agarose gel electrophoresis.

A genomic assay was then developed for each patient on the basis of breakpoint sequence [29,42]. The Custom TaqMan technology (Applied Biosystems by Life Technology, Monza, Italy) was used to detect BCR-ABL1. Patient-specific fusion sequences, all intronic, were submitted to the RepeatMasker program (Institute for System Biology http://www.repeatmasker.org) to span repetitive elements. Primer Express software (Applied Biosystems by Life Technology, Monza, Italy) was used to design common primer forward and MGB-probe located in BCR, and 2 different primers reverse, one located in ABL and one in BCR, used as control (Supplemental Table S3).

The reactions were set up in 96-well Fast Optical Reaction plates using 12.5μl of the TaqMan® Universal PCR Master Mix (Applied Biosystems by Life Technology, Monza, Italy), 600 nM of each primer, 200 nM of custom MGB-probe, 200 ng of gDNA and nuclease free water in a final volume of 25 μl. Reactions were prepared and run in triplicate on an ABI Prism 7000 (Applied Biosystems by Life Technology, Monza, Italy), and each experiment was repeated and confirmed from once to eleven times. The Q-PCR thermal profile was 2 minutes at 50°C, 10 minutes at 95°C and 45 amplifications cycles of 15 seconds at 95°C followed by 1 minute at 60°C.

### Specificity and sensitivity of patient-specific gDNA Q-PCR assays

We evaluated the sensitivity and the efficiency of the Q-PCR assay by performing serial 10-fold dilutions of the leukemic DNA from K562 cells in G147A commercial human male genomic DNA (BCR-ABL1 negative) (Promega, Madison, Wisconsin, USA) to simulate different concentrations of leukemic DNA. We achieved the sensitivity of 10-5.

The dilutions were tested in 6 replicate reactions and a standard curve was obtained by plotting the logarithmic value of the dilution (x-axis) against the average cycle threshold (Ct) of the reactions at each dilution (y-axis). The efficiency of the reaction was calculated as η=(10^−1^/^slope^-1)*100.

The specificity of the patients specific Q-PCR reactions was tested using at least 2 different BCR-ABL1 positive gDNA samples from patients or cell lines, and the efficiency and the sensitivity of each assay was tested using serial 10-fold dilutions of plasmid containing the BCR-ABL1 breakpoint of each patient starting from 400 ng of DNA [29].

We defined a “quantitative range” of detection, the portion of the standard curve in which the minimal residual disease (MRD) levels can be quantified reproducibly and accurately, and we defined the “limit of sensitivity”, the lowest MRD level that still can be detected, although not in all replicates [43]. We thus calculated the exact number of leukemic cells only when the MRD fell within the range of quantization. The detection of MRD at the limit of sensitivity achieved was indicated as positive but not quantified.

### Leukemic cells number calculation

We developed a formula to calculate the percentage of the leukemic cells (LC): %LC= (2/(2^Δct^+1))*100, where ΔCt is the difference between the amplification cycles of the BCR-ABL1 and BCR reactions [29].

Each sample was tested in 6 to 19 replicates, so that we analyzed an average of 2.5 μg of total DNA (from 1.2 μg to 3.8 μg) using 200 ng of DNA in each reaction. In pt.3 at 89th follow-up we analyzed a total of 10.8 μg of DNA in 36 replicates. Assuming that the content of DNA per single cell is 5.7 pg,35 the total number of cells analyzed was calculated by dividing the total amount of DNA analyzed in each reaction by 5.7 pg.

The number of LC was then calculated by multiplying the total number of cells in each sample for the percentage of LC calculated by the ΔCt formula. We thus calculated the exact number of leukemic cells only when the MRD fell within the range of quantization.

The detection of MRD at the limit of sensitivity was indicated as positive but not quantified, leaving the interpretation of the data to clinical judgment (Supplemental Table S1).

### Statistical analysis

The statistical significance of differences was assessed with the Student t test using GraphPad software.

### Flow cytometry

Fresh mononuclear cells were isolated from anti-coagulated bone marrow using Ficoll-Paque density gradient centrifugation. CD34+ cells were isolated using a CD34 MicroBeads Kit (# 130-046-702) and the autoMACS automated selection device (MiltenyiBiotec, Bergisch Galdbach, Germany) according to the manufacturer's instructions. Purity, assessed by flow cytometry, was always >65%.

## SUPPLEMENTARY TABLES


