# Supplementary material for: Genomic quantitative real-time PCR proves residual disease positivity in more than 30% samples with negative mRNA-based qRT-PCR in Chronic Myeloid Leukemia
Source: Oncoscience. 2014 Jul 23;1(7):510–21. doi: 10.18632/oncoscience.65 (PMC4278316; doi:10.18632/oncoscience.65)
Supplement: Supplementary file 1 [file oncoscience-01-0510-s001.pdf]

Genomic quantitative real-time PCR proves residual disease positivity in more than 30 % samples with negative mRNA-based qRT-PCR in chronic myeloid leukemia

Supplemental Table S1: Minimal residual disease detected by hematologic analysis, cytogenetic techniques, qRT-PCR based on mRNA and Q-PCR based on gDNA. Eight CML patients were the object of our study. We monitored minimal residual disease by both hematologic analysis, CBA and I-FISH, and molecular techniques in 8 years of follow-up. Conventional qRT- PCR was performed. The BCR-ABL1/ABL1 ratios were multiplied by the conversion factor of the Bergamo laboratory to set the results on international scale (IS). The same samples were tested by Q-PCR based on genomic DNA. Patient specific Q-PCR assays were developed on the basis of genomic breakpoint and the percentage of leukemic cells was calculated by using the following formula: $\%LC= (2/(2^{\Delta ct}+1))*100$, where ΔCt is the difference between the amplification cycles of the BCR-ABL1 and BCR reactions. The number of LC was then calculated by multiplying the total number of cells analyzed in each sample for the percentage of LC calculated by the ΔCt formula. Assuming that the content of DNA per cell is 5.7 pg, the total analyzed cell number was calculated by dividing the total of pg of gDNA analyzed in each reaction of real-time by 5.7 pg. The detection of MRD at the limit of sensitivity was indicated as positive (0.001 and +) but not quantified. ND (not determined), NE (not evaluated).

Pt. 1				Levels of residual disease (%)		Ph^{+} cells detected by DNA Q-PCR		
Follow-ups (Months)	White blood cell count ($*10^{9}$ cells/L)	CBA	FISH	BCR-ABL1/ABL1 mRNA % (IS)	Leukemic cells DNA (%)	Leukemic cells number	Total analized cell number	Therapy with Gleevec mg/day
1	n.d.	ND	ND	89.002	36.173	76154	210000	400
3	3.6			2.73	0.077	162	210000	400
7	4.6	normal (20 metaphases)	normal (300 nuclei/15 metaphases)	0.05	0.004	8	210000	400
30	3.62	normal (22 metaphases)	ND	0.004	0.001	+	421000	400
33	3			0	0.001	+	421000	400
36	3.69			0.025	0.001	+	631000	400
38	2,99			0	0,001	+	421000	400
41	4.08			0.003	0.001	+	421000	400
50	3.37			0	0.001	+	421000	400
53	3.74			0	0.001	+	631 000	400
59	3.76			0	0.001	+	526000	400
65	3.64			0	0.001	+	526000	400
71	n.d.			0	0.001	+	526000	400
77	n.d.			0	0	0	526000	400
85	n.d.			0	0	0	526000	400

Pt. 2				Levels of residual disease (%)		Ph+ cells detected by DNA Q-PCR		
Follow-ups (Months)	White blood cell count (*10^9 cells/L)	CBA	FISH	BCR-ABL1/ABL1 mRNA % (IS)	Leukemic cells DNA (%)	Leukemic cells number	Total analized cell number	Therapy with Gleevec mg/day
0	54.48	46,XY, t(9;22)(q34;q11) (20 metaphases)	positivity for t(9;22) (300 nuclei)	100	90.536	190602	210000	0
6	3.66	normal (20 metaphases)	normal (300 nuclei)	0.92	0.801	1686	210000	400
15	3.26			0.2	0.079	166	210000	400
18	4.21	normal (22 metaphases)	normal (300 nuclei/10 metaphases)	0.18	0.061	128	210000	400
21	3.54			0.06	0.027	57	210000	400
39	3.73			0.009	0.013	42	315000	400
42	3.13			0.012	0.011	33	315000	400
45	4			0	0.001	+	315000	400
48	4.12	normal (22 metaphases)	normal (300 nuclei/8 metaphases)	0.002	0.004	13	315000	400
51	3.7			0	0.001	+	210000	400
54	4.2			0	0.001	+	315000	400
60	4.47			0	0.001	+	421000	400
67	4.62			0	0.001	+	315000	400
88	n.d.			0	0.001	+	526000	400
94	n.d.			0	0	0	526000	400

Pt. 3				Levels of residual disease (%)		Ph+ cells detected by DNA Q-PCR		
Follow-ups (Months)	White blood cell count (*10^9 cells/L)	CBA	FISH	BCR-ABL1/ABL1 mRNA % (IS)	Leukemic cells DNA (%)	Leukemic cells number	Total analized cell number	Therapy with Gleevec mg/day
0	n.d.	46,XX, t(9;22;16)(q34;q11;q24) (20 metaphases)	Positivity for BCR/ABL1 (der22 and der 16) (320 nuclei)	100	60.508	127384	210000	0
3	n.d.			11.006	2.228	4691	210000	400
6	4	normal (20 metaphases)	normal (300 nuclei)	0.712	0.032	66	210000	400
12	3.35	normal (23 metaphases)	normal (300 nuclei/15 metaphases)	0.004	0.016	34	210000	400
15	3.41			0.811	0.009	19	210000	400
18	3.94	normal (23 metaphases)	normal (300 nuclei/15 metaphases)	0.016	0.006	13	210000	400
21	4.14			0.023	0.005	11	210000	400
27	3.97			0.011	0.003	6	210000	400
30	2.96	normal (22 metaphases)	normal (400 nuclei)	0	0.002	4	210000	400
42	n.d.			0	0.001	+	210000	400
48	3.2	normal (20 metaphases)	normal (300 nuclei/10 metaphases)	0	0	0	210000	400
55	4.3			0.026	0.001	+	210000	400
58	3.8			0	0.001	+	245000	400
64	3.5			0	0.001	+	210000	400
76	4.2			0	0	0	526000	400
82	3.6			0	0	0	526000	400
89	n.d.			0	0.001	+	1894000	400
95	n.d.			0	0	0	526000	400
98	n.d.			0	0	0	526000	400

Pt. 4				Levels of residual disease (%)		Ph+ cells detected by DNA Q-PCR		
Follow-ups<br><br>(Months)	White blood<br><br>cell count (*10^9 cells/L)	CBA	FISH	BCR-ABL1/ABL1<br><br>mRNA % (IS)	Leukemic cells<br><br>DNA (%)	Leukemic cells<br><br>number	Total analized<br><br>cell number	Therapy with Gleevec<br><br>mg/day
0	175	46,XY, t(9;22)(q34;q11) (20 metaphases)	positivity for t(9;22) (200 nuclei/20 metaphases)+del ABL der(9)	64.528	90	189473	210000	0
6	6.98	normal (20 metaphases)	normal (300 nuclei)	0.35	0.883	1859	210000	800
12	7.11	normal (24 metaphases)	normal (300 nuclei/30 metaphases)	0.23	0.204	429	210000	800
16	8.43			0.14	0.128	269	210000	800
20	6.99	normal (21 metaphases)	normal (400 nuclei/30 metaphases)	1.06	0.533	1122	210 000	800
22	6.88			0.02	0.096	202	210000	800
32	6.49	normal (20 metaphases)	normal (300 nuclei/5 metaphases)	0.26	0.678	1427	210000	800
44	6.91			0	0.001	+	315000	800
47	7.51			0	0.001	+	315000	800
58	7.39			0	0.001	+	315000	800
64	7.13			0	0.001	+	315000	800
71	6.41			0.003	0.001	+	315000	400
77	7.63			0	0.001	+	421000	400
83	6.4			0	0.001	+	526000	400
89	n.d.			0	0.001	+	526000	400
95	n.d.			0	0.001	+	526000	400
101	n.d.			0	0	0	526000	400

Pt. 5				Levels of residual disease (%)		Ph^+ cells detected by DNA Q-PCR		
Follow-ups (Months)	White blood cell count ($*10^9$ cells/L)	CBA	FISH	BCR-ABL1/ABL1 mRNA % (IS)	Leukemic cells DNA (%)	Leukemic cells number	Total analized cell number	Therapy with Gleevec mg/day
0	68.7	46,XY, t(9;22)(q34;q11) (20 metaphases)	positivity for t(9;22) (300 nuclei)	100	93	195789	210000	0
13	4.1	normal (20 metaphases)	normal (300 nuclei/4 metaphases)	0.04	0.007	15	210000	400
19	3.95	normal (21 metaphases)	normal (300 nuclei/4 metaphases)	0.05	0.012	25	210000	600
35	3.7			0.089	0.001	+	315000	600
44	3.37			0.09	0.001	+	315000	600
51	5.7			0	0.005	15	315000	400
57	4.2			0	0.001	+	315000	400
63	3.7			0	0.001	+	315000	400
70	5.3			0	0.013	40	315000	400
75	4.5			0	0.001	+	315000	400
89	n.d.			0	0.001	+	526000	400
95	n.d.			0	0.001	+	526000	400

Pt. 6				Levels of residual disease (%)		Ph+ cells detected by DNA Q-PCR		
Follow-ups (Months)	White blood cell count (*10^9 cells/L)	CBA	FISH	BCR-ABL1/ABL1 mRNA % (IS)	Leukemic cells DNA (%)	Leukemic cells number	Total analized cell number	Therapy with Gleevec mg/day
0	n.d.	46,XY, t(9;22)(q34;q11) (20 metaphases)	positivity for t(9;22) (300 nuclei)	100	88.993	187353	210000	0
4	280			7.07	4.575	9632	210000	400
7	4.9	normal (20 metaphases)	normal (300 nuclei)	1.45	0.672	1415	210000	400
13	6.1	normal (20 metaphases)	normal (300 nuclei)	0.58	0.418	880	210000	400
16	6.6			1.66	0.032	67	210000	400
19	5.1	normal (23 metaphases)	normal (300 nuclei/8 metaphases)	0.45	0.045	95	210000	400
22	5.7			0.4	0.068	143	210000	400
25	5.9	normal (20 metaphases)	normal (300 nuclei/5 metaphases)	0.32	0.024	51	210000	400
37	6.3			0.023	0.006	18	315000	400
51	6.6			0	0.001	+	421000	400
57	7.4			0	0.005	17	315000	400
63	4.7			0	0.008	24	315000	400
69	9.8			0.003	0.005	17	315000	400
84	n.d.			0	0.001	+	526000	400
90	n.d.			0	0.006	31	526000	400
94	n.d.			N.E.	0.001	+	526000	400
97	n.d.			0	0.001	+	526000	400

Pt. 7				Levels of residual disease (%)		Ph+ cells detected by DNA Q-PCR		
Follow-ups (Months)	White blood cell count (*10^9 cells/L)	CBA	FISH	BCR-ABL1/ABL1 mRNA % (IS)	Leukemic cells DNA (%)	Leukemic cells number	Total analized cell number	Therapy with Gleevec mg/day
0	n.d.	46,XY, t(9;22)(q34;q11) (20 metaphases)	positivity for t(9;22) (300 nuclei)	102.999	90.258	190016	210000	0
3	4.4			0.634	1.064	2240	210000	400
6	n.d.	t(9;22)(q34;q11) (1 methaphase); normal (21 metaphases)	normal (300 nuclei/10 metaphases)	0.071	0.391	823	210000	400
30	6			0.04	0.002	5	280000	400
37	6.7			0	0.001	+	280000	400
43	6.92			0.011	0.001	+	666000	400
47	n.d.			0	0.001	+	280000	400

Pt. 8				Levels of residual disease (%)		Ph+ cells detected by DNA Q-PCR		
Follow-ups (Months)	White blood cell count (*10^9 cells/L)	CBA	FISH	BCR-ABL1/ABL1 mRNA % (IS)	Leukemic cells DNA (%)	Leukemic cells number	Total analizd cell number	Therapy with Gleevec mg/day
0	n.d.	46,XX, t(9;22)(q34;q11) (20 metaphases)	positivity for t(9;22) (300 nuclei/20 metaphases)	35.947	84.079	177008	210000	0
3	2.73			12.48	19.669	41408	210000	400
6	4.2	normal (20 metaphases)	normal (300 nuclei)	7.77	4.895	10305	210000	400
12	4.35	t(9;22)(q34;q11) (1 methaphase); normal (22 metaphases)	positivity for t(9;22) (3 nuclei); normal (497 nuclei/20 metaphases)	3.64	3.401	7160	210000	400
18	3.74	normal (21 metaphases)	normal (350 nuclei/5 metaphases)	2.19	1.478	3112	210000	600
21	4.06			7.03	3.567	7509	210000	600
24	4.12	normal (21 metaphases)	normal (400 nuclei/15 metaphases)	1.26	0.855	1800	210000	600
33	4.55			3.88	1.026	2160	210000	600
39	4.58			3.34	0.527	1664	315000	600
43	4.38	normal (20 metaphases)	normal (300 nuclei/15 metaphases)	1.921	0.597	1884	315000	600
46	3.79			0.272	0.583	1841	315000	600
52	4.59			0.36	0.377	1190	315000	600
55	4.9			0.474	0.590	1864	315000	600
58	5.64			0.216	0.742	2345	315000	600
61	5.6			0.354	0.429	1353	315000	600
64	4.83			0.427	0.453	1431	315000	600
67	4			0.703	0.381	1203	315000	600
70	5.4			0.087	0.119	376	315000	600
73	n.d.			0.253	0.098	310	315000	600
79	2.64			0.452	0.174	551	315000	400
83	n.d.			0.26	0.235	1235	526000	400
84	n.d.			0.26	0.068	359	526000	400
86	n.d.			0.495	0.364	1914	526000	400
89	n.d.			0.61	0.240	1263	526000	400
91	n.d.			0.551	0.040	208	526000	400
94	n.d.			0.475	0.233	1224	526000	400

Supplemental Table S2: Breakpoint features. Breakpoints were positioned according to the reference sequence of chromosome 22 (NC_000022) and chromosome 9 (NC_000009).

	Breakpoint position	
ID	Chr. 22	Chr. 9
1	21962041	132640551
2	21962170	132568941
3	21962142	132584258
4	21962501	132709774
5	21962360	132580996
6	21961956	132580831
7	21961917	132580474
8	21962256	132604043
K562	21962742	132596973

Supplemental table S3: Primers and probe for the gDNA Q-PCR.

ID	
1	F 5'-CTGCTGCTGGGTGGTTGA-3'<br>Ph-R 5'-GGATTTTAGTCCTTACTTGTTTCCTATTTCAC-3'<br>wt-R 5'-GCCAGATCCAAGGCACAGA-3'<br>Probe 6-FAM-AGATGCACGGCTTG-MGB
2	F 5'-GCCCTCCTCTCCTCCAGCTA-3'<br>Ph-R 5'-AAGCCTCTGGCGTGTTTCC-3'<br>wt-R 5'-TGAGCATATGTGCAACAGTGAATG-3'<br>Probe 6-FAM-CACTTTTGGTCAAGCTG-MGB
3	F 5'-TGCTCTGTGCCTTGGATCTG-3'<br>Ph-R 5'-TTCGGTGTAAAATCCTTCCATACTTT-3'<br>wt-R 5'-TGCAAAACAGCTTGACCAAAA-3'<br>Probe 6-FAM-CCCCACTCCCGTCCT-MGB
4	F 5'-TTGTCACCTGCCTCCCTTTC-3'<br>Ph-R 5'-TGAACTCCTGACCTCAAGTGATCT-3'<br>wt-R 5'-GAGCCCCGGAGACTCATCA-3'<br>Probe 6-FAM-CGGGACAACAGAAGC-MGB
5	F 5'-CACTGGTTTGCCTGTATTGTGAA-3'<br>Ph-R 5'-GGACACACAGGGAACTACACTGC-3'<br>wt-R 5'-TGGGCCAAAAACATACTCATCA-3'<br>Probe 6-FAM-TCCTGAGATCCCC-MGB
6	F 5'-TGGGACTAGTGGACTTTGGTTCA-3'<br>Ph-R 5'-GTGCATGATCATCACTAGTTAAAATGTAAA-3'<br>wt-R 5'-CTAACCCACCTTGTCCACTCCT-3'<br>Probe 6-FAM-ACAAGAGGCCCTAACAA-MGB
7	F 5'-CCCCCTTCCTGTTAGCACTTT-3'<br>Ph-R 5'-GCTGCAACAGTACAAACAGTAACCC-3'<br>wt-R 5'-CCCTAACAAGCATAGCTCTTGCTT-3'<br>Probe 6-FAM-ATGGGACTAGTGGACTTT-MGB
8	F 5'-CACAGCATACGCTATGCACATGT-3'<br>Ph-R 5'-GGGAAAAAAATGTTTTCTCCTTATATCG-3'<br>wt-R 5'-ATAAGGTTCCAAGCACAGCAGAG-3'<br>Probe 6-FAM-ACACACACCCCACCC-MGB
K562	F 5'-TGACCACGGGACACCTTTG-3'<br>Ph-R 5'-TGGGTATGGAAGCTGATAAAACC-3'<br>Probe 6-FAM-CCCTGGCCGCTGTG-MGB
